# Synthesis of [(4-Chloro-5*H*-1,2,3-dithiazol-5-ylidene)amino]azines

**DOI:** 10.3390/molecules16118992

**Published:** 2011-10-25

**Authors:** Panayiotis A. Koutentis, Maria Koyioni, Sophia S. Michaelidou

**Affiliations:** Department of Chemistry, University of Cyprus, P.O. Box 20537, 1678 Nicosia, Cyprus; Email: maria_koyioni@hotmail.com (M.K.); sophie_michaelidou@hotmail.com (S.S.M.)

**Keywords:** heterocycle, Appel salt, 1,2,3-dithiazoles, condensation, azine

## Abstract

The reactions of 2-, 3- and 4-aminopyridines with 4,5-dichloro-1,2,3-dithiazol-ium chloride (Appel salt) **4** to give *N*-(4-chloro-5*H*-1,2,3-dithiazol-5-ylidene)pyridin-X-amines **1a** (X = 2), **1g** (X = 3) and **1k** (X = 4) were optimized with respect to base, temperature and reaction time. Based on these conditions a total of thirteen [(dithiazol-ylidene)amino]azines **1a-m** were prepared and fully characterized.

## 1. Introduction

Select *N*-(4-chloro-5*H*-1,2,3-dithiazol-5-ylidene)anilines (*N*-aryl-1,2,3-dithiazol-5*H*-imines) show interesting antitumor [1], antibacterial [2,3,4], antifungal [5,6,7], and herbicidal [8] activity. The biological activity could be due to the 1,2,3-dithiazole ring, that acts as an inhibitor of several enzymes that are structurally related to serine proteases [9]. Furthermore, *N*-aryldithiazolimines are useful precursors to other heterocycles. For example the thermolysis of *N*-aryldithiazolimines can afford benzothiazoles [10,11], benzimidazoles [12], thiazolopyridines [13] and benzoxazines [14]. Moreover, *N*-aryldithiazolimines can also be transformed into useful acyclic functionalities such as cyanothio-formanilides [15,16,17], *N*-arylcyanoformimidoyl chlorides [10,18] and *N*-arylisothiocyanates [19,20].

**Figure 1:**
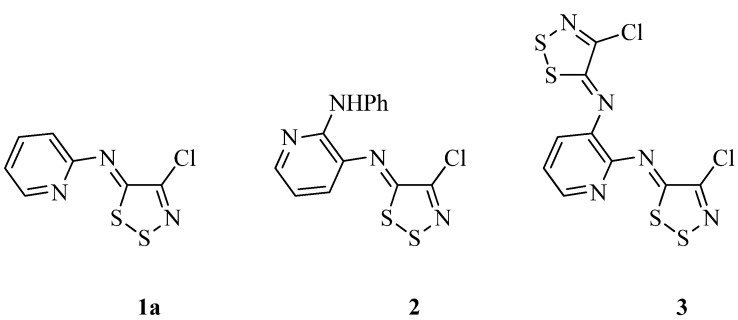
Structures of known *N*-(4-chloro-5*H*-1,2,3-dithiazol-5-ylidene)pyridinamines.

Despite their synthetic utility the library of dithiazolimines prepared from Appel salt **4** is comprised mainly of analogues where the dithiazolimine moiety is bound to a benzene ring. Some examples also exist where the dithiazole moiety is bound to 5-membered heteroles [21,22,23], but to the best of our knowledge the only reported examples, where the dithiazolimine moiety is bound directly to a 6-membered azine, are *N*-(4-chloro-5*H*-1,2,3-dithiazol-5-ylidene)pyridin-2-amine (**1a)** [24], *N*^3^-(4-chloro-5*H*-1,2,3-dithiazol-5-ylidene)-*N*^2^-phenylpyridine-2,3-diamine (**2**) [25] and *N*^2^,*N*^3^-bis(4-chloro-5*H*-1,2,3-dithiazol-5-ylidene)pyridine-2,3-diamine (**3**) [25] (Figure 1).

This may be due to the fact that electron poor aminoazines, such as aminopyridines, have less nucleophilic character than primary anilines. This can lead to low yields of the desired [(4-chloro-5*H*-1,2,3-dithiazolylidene)amino]azines and/or complex reaction mixtures due to side reactions [26]. As such, it was decided to study the synthesis of the less explored [(4-chloro-5*H*-1,2,3-dithiazol-ylidene)amino]azines. Herein we wish to report our results.

## 2. Results and Discussion

### 2.1. Studies on N-(4-Chloro-5H-1,2,3-dithiazol-5-ylidene)pyridinamines

**Scheme 1:**
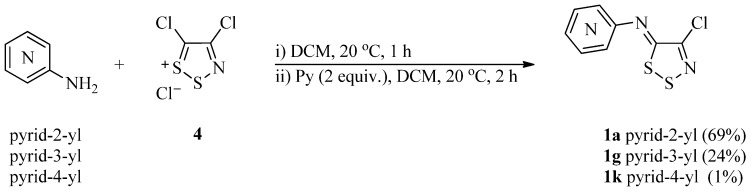
Preparation of *N*-(4-chloro-5*H*-1,2,3-dithiazol-5-ylidene)pyridinamines **1a**, **1g** and **1k**.

Our investigation began with the reaction of the simplest aminoazines, namely aminopyridines, with Appel salt **4** to afford *N*-(4-chloro-5*H*-1,2,3-dithiazolylidene)pyridin-X-amines (where X = 2, 3 and 4). By focusing of the 2-, 3- and 4-aminopyridines we hoped to see how the position of the pyridyl ring nitrogen affected the reactivity of the aminoazine with Appel salt **4**. These reactions were optimized with respect to base, temperature, and reaction time (Scheme 1).

Initially the synthesis of *N*-(4-chloro-5*H*-1,2,3-dithiazol-5-ylidene)pyridinamines **1a**, **1g** and **1k** was based on a well established literature procedure for the preparation of (dithiazolylideneamino)arenes [24], using pyridine (2 equiv.) as base, at *ca.* 20 °C in DCM (Scheme 1). It became clear even at this stage that the product yields were affected by the position of the pyridyl nitrogen: The reaction between 2-, 3- and 4-aminopyridines and Appel salt **4** gave the corresponding *N*-(4-chloro-5*H*-1,2,3-dithiazol-5-ylidene)pyridin-X-amines **1a** (X = 2), **1g** (X = 3) and **1k** (X = 4) in 69, 24 and 1% yields, respectively.

To improve the yields in the above reactions we then screened a variety of amine bases. Weakly aromatic amine bases like pyridine (p*K*_b_ 8.8) and the more sterically demanding (less nucleophilic) lutidine (p*K*_b_ 7.4) were included, as well as a range of trialkylamines with increasing steric demands, reduced nucleophilicity and increasing basicity e.g., DABCO (p*K*_b_ 5.2), Et_3_N (p*K*_b_ 3.4), and Hünig’s base (p*K*_b_ 2.6), and “weakly” nucleophilic strong amidine bases such as DBU (p*K*_b_ 1.1) and DBN (p*K*_b_ 0.5) [27].

The addition of base was needed to obtain greater than trace quantities of (dithiazolylidene)-pyridinamines. Furthermore, increasing the reaction time (by 4, 6 and 8 h) before or after the addition of base, decreased the yields. Increasing the reaction temperature from 25 to 40 °C also did not lead to an improvement of the observed yields. The best conditions required mixing Appel salt **4** with the aminopyridine for 1 h at room temperature followed by the addition of amine base (2 equiv.) and a further 2 h of stirring (Table 1).

**Table 1:** Reaction of Appel salt **4** (2.18 mmol) with aminopyridines (2.18 mmol) in DCM (4 mL) at *ca.* 20 °C for 1 h and followed by base (4.36 mmol) for another 2 h.

Aminopyridine	Base (p*K*_b_)	Yields of 1a, 1g or 1k (%)
Pyridin-2-amine	Pyridine (8.8)	**1a** (69)
	2,6-Lutidine (7.4)	**1a** (72)
	Dabco (5.2)	**1a** (55)
	Et_3_N (3.4)	**1a** (69)
	i-Pr_2_NEt (2.6)	**1a** (73)
	DBU (1.1)	**1a** (58)
	DBN (0.5)	**1a** (47)
Pyridin-3-amine	Pyridine (8.8)	**1g** (24)
	2,6-Lutidine (7.4)	**1g** (45)
	Dabco (5.2)	**1g** (8)
	Et_3_N (3.4)	**1g **(43)
	i-Pr_2_NEt (2.6)	**1g **(57)
	DBU (1.1)	**1g** (16)
	DBN (0.5)	**1g** (13)
Pyridin-4-amine	Pyridine (8.8)	**1k** (traces)
	2,6-Lutidine (7.4)	**1k** (traces)
	Dabco (5.2)	**1k** (3)
	Et_3_N (3.4)	**1k** (23)
	i-Pr_2_NEt (2.6)	**1k** (13)
	DBU (1.1)	**1k** (13)
	DBN (0.5)	**1k** (traces)

**Scheme 2:**
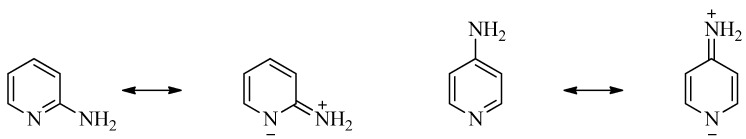
Zwitterionic resonance forms for 2- and 4-aminopyridines.

The highest yields of *N*-(4-chloro-5*H*-1,2,3-dithiazol-5-ylidene)pyridin-2-amine (**1a**) and *N*-(4-chloro-5*H*-1,2,3-dithiazol-5-ylidene)pyridin-3-amine (**1g**), 73 and 57%, respectively, were obtained with Hünig’s base, while the highest yield for *N*-(4-chloro-5*H*-1,2,3-dithiazol-5-ylidene)pyridin-4-amine (**1k**) (23%) was obtained with Et_3_N. The exceptionally low yields of product from the reaction of 4-aminopyridine and Appel salt **4** may be partially explained by the reduced nucleophilicity of the primary amine, which is due to a significant contribution of a zwitterionic resonance form. This deactivation is not observed with 2-aminopyridine since the contribution of the analogous zwitterionic resonance form is considerably less important (Scheme 2) [28]. Moreover, 4-aminopyridine was not very soluble in the solvents tried (DCM, PhH, MeOH, EtOH,) and this could also have contributed to the low product yields.

**Scheme 3:**
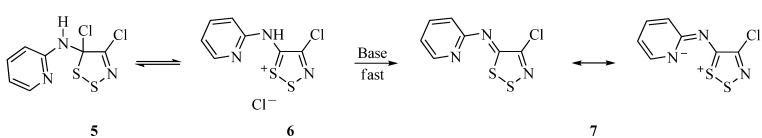
Proposed intermediates for the synthesis of (dithiazolylidene)pyridin-2-amine **7**.

Also notable was that the reaction between 2-aminopyridine and Appel salt **4** was less sensitive to the base used, which tentatively may be attributed to two factors: (1) The pyrid-2-yl nitrogen’s ability to coordinate with the dithiazole sulfur S-1 in a “non-bonding” manner [29,30] provided particularly stable (dithiazolylidene)pyridinamines; and (2) the acidity of the proton in intermediate **6** was enhanced by both the neighbouring pyridyl nitrogen and the positively charged dithiazolium ring sulfur [9,23]. Both these features could lead to a very facile base catalysed elimination of HCl (Scheme 3).

### 2.2. Synthesis of a [(4-Chloro-5*H*-1,2,3-dithiazol-5-ylidene)amino]azine Library

To investigate this further, a range of substituted aminopyridines and related azines were reacted with Appel salt **4** in the presence of the above amine bases (Table 2). In nearly all cases the substituents (Me, Hal, CN, and NO_2_) on the aminoazine had little effect on the product yields. The exception to this was the use of 3-aminopyridin-2-one, which only gave good yields (53%) of the dithiazolylideneamine **1h** when pyridine was used as base. As before, the position of the nitrogen atom in the aromatic ring affected the product yields. In the case of 2- and 3-amino derivatives the desired [(dithiazolylidene)amino]azines were obtained in moderate to good yields. 4-Aminopyridines gave very low yields with all the bases as expected; however, the presence of an additional nitrogen atom *α* to the amine as in 4-aminopyrimidine-5-carbonitrile led to the formation of the dithiazolylideneamine **1m** in moderate to good yields (15–61%).

**Table 2:** Reaction of 4,5-dichloro-1,2,3-dithiazolium chloride 4 (2.18 mmol) with aminoazines (2.18 mmol) in DCM (4 mL) at *ca.* 20 °C for 1 h and then addition of base (4.36 mmol) for 2 h to give [(4-chloro-5*H*-1,2,3-dithiazolylidene)amino]azines **1a–m**.

Azine	Product Yields (%)						
	Pyridine	Lutidine	DABCO	Et_3_N	i-Pr_2_NEt	DBU	DBN
**1a** (pyrid-2-yl)	69	72	55	69	73	58	47
**1b** (3-MeO-pyrid-2-yl)	71	67	55	60	66	42	40
**1c** (3,5-Cl_2_-pyrid-2-yl)	69	70	52	44	43	44	39
**1d** (3,5-Br_2_-pyrid-2-yl)	65	70	56	48	40	27	15
**1e** (3-O_2_N-pyrid-2-yl)	45	62	48	32	14	23	12
**1f** (pyrazin-2-yl)	65	63	51	63	61	40	36
**1g** (pyrid-3-yl)	23	45	8	42	57	16	10
**1h** (2-HO-pyrid-3-yl)	53	19	11	9	18	12	5
**1i** (2-Cl-pyrid-3-yl)	75	82	85	71	72	50	45
**1j** (4-Cl-pyrid-3-yl)	65	76	60	70	69	21	19
**1k** (pyrid-4-yl)	trace	trace	3	21	13	13	10
**1l **(2,6-Me_2_-pyrid-4-yl)	5	trace	19	22	10	9	5
**1m** (5-NC-pyrimid-4-yl)	61	55	35	45	40	39	15

## 3. Experimental

### 3.1. General

Reactions were protected from atmospheric moisture by CaCl_2_ drying tubes. Anhydrous Na_2_SO_4_ was used for drying organic extracts, and all volatiles were removed under reduced pressure. All reaction mixtures and column eluents were monitored by TLC using commercial glass backed thin layer chromatography (TLC) plates (Merck Kieselgel 60 F_254_). The plates were observed under UV light at 254 and 365 nm. The technique of dry flash chromatography was used throughout for all non-TLC scale chromatographic separations using Merck Silica Gel 60 (less than 0.063 mm). Melting points were determined using a PolyTherm-A, Wagner & Munz, Koefler–Hotstage Microscope apparatus. Solvents used for recrystallization are indicated after the melting point. UV spectra were obtained using a Perkin-Elmer Lambda-25 UV/vis spectrophotometer and inflections are identified by the abbreviation ‘inf’. IR spectra were recorded on a Shimadzu FTIR-NIR Prestige-21 spectrometer with a Pike Miracle Ge ATR accessory and strong, medium and weak peaks are represented by s, m and w, respectively. ^1^H- and ^13^C-NMR spectra were recorded on a Bruker Avance 300 instrument (at 300 and 75 MHz, respectively). ^13^C-DEPT NMR was used to identify quaternary and tertiary carbons, which are indicated by (s) and (d) notations, respectively. Deuterated solvents were used for homonuclear lock and the signals are referenced to the deuterated solvent peaks. Low resolution (EI) mass spectra were recorded on a Shimadzu Q2010 GC-MS with direct inlet probe.

### 3.2. General Procedure for the Synthesis of [(4-Chloro-5*H*-1,2,3-dithiazol-5-ylidene)amino]azines

To a stirred solution of 4,5-dichloro-1,2,3-dithiazolium chloride (**4**, 454.2 mg, 2.18 mmol) in DCM (4 mL) at *ca*. 20 °C and protected with CaCl_2_ drying tube, was added the corresponding aminoazine (1 equiv., 2.18 mmol). After 1 h, to the reaction mixture was added, dropwise, the appropriate base (2 equiv., 4.36 mmol) and the mixture left to stir at *ca*. 20 °C for additional 2 h. The reaction mixture was subsequently adsorbed onto silica and chromatographed to afford the corresponding [(4-chloro-5*H*-1,2,3-dithiazol-5-ylidene)amino]azine **1**.

*(Z)-N-(4-Chloro-5H-1,2,3-dithiazol-5-ylidene)pyridin-2-amine* (**1a**): Yellow-green needles (365.6 mg, 73%), mp 149–150 °C (lit. [24], 154–155 °C) (cyclohexane); Anal. found: C, 36.6; H, 1.7; N, 18.3. C_7_H_4_ClN_3_S_2_ requires C, 36.6; H, 1.8; N, 18.3%); UV *λ*_max_(DCM)/nm 246 (log *ε* 2.83), 294 (2.48), 388 (2.84), 405 (2.96), 427 (2.76); IR *v*_max_/cm^−1^1589w, 1560w, 1512m, 1491m, 1449m, 1431m, 1296w, 1267w, 1258w, 1175m, 1142m, 1092w, 1042w, 999w, 891m, 862m, 787s, 742m, 704m; ^1^H-NMR (CDCl_3_) *δ*_H_ 8.60 (1H, d, *J* 4.2, *H*-3 or 6), 7.90 (1H, ddd, 7.7, 7.7, 1.7, *H-*4 or 5), 7.67 (1H, d, *J* 8.1, *H*-3 or 6), 7.28 (1H, ddd, *J* 7.2, 5.1, 1.0, *H*-4 or 5); ^13^C-NMR (CDCl_3_) *δ*_C_ 157.9 (s), 153.9 (s), 148.9 (s), 143.3 (d), 138.4 (d), 122.4 (d), 121.6 (d); MS *m/z* (EI) 231 (M^+^+2, 8%), 229 (M^+^, 19), 194 (85), 168 (5), 162 (5), 125 (3), 104 (3), 78 (100), 70 (6), 64 (20), 51 (43).

*(Z)-N-(4-Chloro-5H-1,2,3-dithiazol-5-ylidene)-3-methoxypyridin-2-amine* (**1b**): Yellow fibers (402 mg, 71%), mp 190–191 °C (cyclohexane/EtOH); Anal. found: C, 36.9; H, 2.1; N, 16.3. C_8_H_6_ClN_3_OS_2_ requires C, 37.0; H, 2.3; N, 16.2%); UV *λ*_max_(DCM) 228 (log *ε* 3.69), 249 (3.70), 304 (3.42), 361 inf (3.05), 374 inf (3.33), 386 inf (3.53), 403 (3.78), 425 (3.85), 449 (3.61); IR *v*_max_/cm^−1^ 3075w, 2970w, 2936w, 2837w, 1572m, 1508m, 1487m, 1464m, 1449w, 1431s, 1425s, 1308s, 1294m, 1279s, 1263w, 1209w, 1182m, 1171m, 1155w, 1125s, 1076w, 1013s, 951w, 893m, 868s, 808s, 783s, 773w, 764s; ^1^H-NMR (DMSO-*d*_6_) *δ*_H_ 8.22 (1H, d, *J* 4.2, *H*-4 or 6), 7.63 (1H, d, *J* 7.8, *H*-4 or 6), 7.43 (1H, dd, *J* 8.1, 4.8, *H*-5), 3.95 (3H, s, C*H*_3_O); ^13^C-NMR (DMSO-*d*_6_) *δ*_C_156.9 (s), 150.6 (s), 148.3 (s), 144.3 (s), 134.3 (d), 123.3 (d), 119.8 (d), 55.9 (*C*H_3_O); MS *m/z* (EI); 261 (M^+^+2, 8%), 259 (M^+^, 19), 226 (8), 224 (37), 195 (5), 160 (36), 134 (7), 123 (8), 108 (13), 93 (14), 78 (100), 70 (12), 64 (30), 51 (20).

*(Z)-3,5-Dichloro-N-(4-chloro-5H-1,2,3-dithiazol-5-ylidene)pyridin-2-amine* (**1c**): Yellow cotton fibers (456 mg, 70%), mp 145–146 °C (from cyclohexane); Anal. found: C, 28.2; H, 0.6; N, 13.9. C_7_H_2_Cl_3_N_3_S_2_ requires C, 28.2; H, 0.7; N, 14.1%); UV *λ*_max_ (DCM) 229 (log *ε* 3.17), 254 (3.30), 261 (3.26), 313 (2.86), 338 inf (3.07), 397 (3.31), 418 (3.42), 440 (3.22); IR *v*_max_/cm^−1^ 3067w, 3049w (Ar CH), 1564m, 1539m, 1524m, 1491m, 1412s, 1377m, 1279m, 1240m, 1225w, 1171m, 1142w, 1121m, 1061m, 924w, 901m, 880s, 810m, 764m, 756m; ^1^H-NMR (DMSO-*d*_6_) *δ*_H_ 8.64 (1H, d, *J* 2.2, *H*-4 or 6), 8.37 (1H, d, *J* 2.2, *H*-4 or 6); ^13^C-NMR (DMSO-*d*_6_) *δ*_C_ 160.3 (s), 150.1 (s), 148.6 (s), 141.6 (d), 138.4 (d), 127.6 (s), 127.5 (s); MS *m/z* (EI); 301 (M^+^+4, 3%) 299 (M^+^+2, 6), 297 (M^+^, 6), 266 (4), 264 (18), 262 (24), 236 (3), 172 (3), 146 (10), 137 (4), 125 (4), 110 (29), 102 (5), 98 (5), 93 (8), 85 (8), 75 (15), 70 (16), 64 (100), 50 (5).

*(Z)-3,5-Dibromo-N-(4-chloro-5H-1,2,3-dithiazol-5-ylidene)pyridin-2-amine* (**1d**): Yellow cubes (591 mg, 70%), mp 174–175 °C (from cyclohexane); Anal. found: C, 21.8; H, 0.5; N, 10.8. C_7_H_2_Br_2_ClN_3_S_2_ requires C, 21.7; H, 0.5; N, 10.8%); UV *λ*_max_ (DCM) 229 (log *ε* 3.08), 257 (3.16), 262 inf (3.11), 316 (2.69), 325 inf (2.59), 365 inf (2.52), 399 (3.16), 419 (3.27), 442 (3.07); UV *v*_max_/cm^−1^ 1539m, 1514m, 1485m, 1454w, 1414s, 1364m, 1314w, 1275m, 1244w, 1231w, 1167m, 1123w, 1098m, 1045m, 914w, 897m, 891m, 876s, 795s, 754m, 737s; ^1^H-NMR (DMSO-*d*_6_) *δ*_H_8.75 (1H, s, *H*-4 or 6), 8.59 (1H, s, *H*-4 or 6); ^13^C-NMR (DMSO-*d*_6_) *δ*_C_ 160.3 (s), 151.1 (s), 148.6 (s), 144.3 (d), 143.9 (d), 118.4 (s), 115.8 (s); MS *m/z* (EI); 391 (M^+^+6, 4%), 389 (M^+^+4, 18), 387 (M^+^+2, 21), 385 (M^+^, 9), 354 (25), 352 (37), 350 (20), 310 (10), 308 (28), 306 (23), 262 (5), 247 (11), 245 (11), 238 (8), 236 (17), 234 (9), 183 (6), 181 (6), 156 (21), 154 (19), 125 (7), 102 (12), 93 (9), 76 (41), 64 (100), 50 (15).

*(Z)-N-(4-Chloro-5H-1,2,3-dithiazol-5-ylidene)-3-nitropyridin-2-amine* (**1e**): Yellow cotton fibers (371 mg, 62%), mp 188–189 °C (from cyclohexane); Anal. found: C, 30.7; H, 1.0; N, 20.4. C_7_H_3_ClN_4_O_2_S_2_ requires C, 30.6; H, 1.1; N, 20.4%); UV *λ*_max_(DCM)/nm 230 (log *ε* 2.93), 278 (2.49), 395 inf (2.90), 411 (3.03), 432 (2.88); IR *v*_max_/cm^−1^ 1597m, 1560m, 1526s, 1479m, 1427s, 1362m, 1344s, 1279w, 1261w, 1244w, 1169m, 1086w, 901s, 878m, 847s, 808s, 770s, 706m; ^1^H-NMR (DMSO-*d*_6_) *δ*_H_ 8.87 (1H, dd, *J* 5.0, 1.5, *H*-6), 8.53 (1H, dd, *J* 7.9, 1.5, *H-*4), 7.59 (1H, dd, *J* 7.9, 5.0, *H*-5); ^13^C-NMR (DMSO-*d*_6_) *δ*_C_ 161.9 (s), 148.6 (s), 148.3 (d), 146.8 (s), 141.6 (s), 133.5 (d), 121.8 (d); MS *m/z* (EI); 276 (M^+^+2, 16%), 274 (M^+^, 37), 239 (18), 228 (41), 226 (99), 210 (4), 191 (6), 155 (12), 149 (35), 137 (13), 119 (78), 102 (18), 91 (63), 76 (38), 70 (29), 64 (100), 50 (22).

*(Z)-N-(4-Chloro-5H-1,2,3-dithiazol-5-ylidene)pyrazin-2-amine* (**1f**): Yellow-orange needles (327 mg, 65%), mp 208–209 °C (from cyclohexane/EtOH); Anal. found: C, 31.2; H, 1.2; N, 24.2. C_6_H_3_ClN_4_S_2_ requires C, 31.2; H, 1.3; N, 24.3%); UV *λ*_max_(DCM) 230 (log *ε* 3.02), 243 (3.02), 252 inf (2.96), 297 (2.72), 322 inf (2.61), 379 inf (2.89), 392 (3.10), 409 (3.23), 430 (3.06); IR *v*_max_/cm^−1^ 1526s, 1501m, 1476s, 1452s, 1406s, 1298m, 1281m, 1179s, 1146m, 1061m, 1016m, 903s, 868s, 845s, 793s, 750w, 714m; ^1^H-NMR (DMSO-*d*_6_) *δ*_H_ 8.95 (1H, d, *J* 1.2, *H*-3), 8.72 (1H, dd, *J* 2.7, 1.5, *H*-5), 8.59 (1H, d, *J* 2.7, *H*-6); ^13^C-NMR (DMSO-*d*_6_) *δ*_C_ 160.6 (s), 150.6 (s), 148.5 (s), 144.7 (d), 141.0 (d), 139.3 (d); MS *m/z* (EI) 232 (M^+^+2, 14%), 230 (M^+^, 32), 195 (100), 169 (11), 125 (12), 102 (8), 93 (6), 84 (6), 79 (70), 70 (16), 64 (68), 52 (66).

*(Z)-N-(4-Chloro-5H-1,2,3-dithiazol-5-ylidene)pyridin-3-amine* (**1g**): Yellow cotton fibers (285 mg, 57%), mp 126–127 °C (from cyclohexane/EtOH); Anal. found: C, 36.7; H, 1.7; N, 18.2. C_7_H_4_ClN_3_S_2_ requires C, 36.6; H, 1.8; N, 18.3%); UV *λ*_max_ (DCM) 231 (log *ε* 2.86), 280 (2.33), 374 (2.65); IR *v*_max_/cm^−1^ 3051w, 3042w, 1570s, 1539w, 1504m, 1474m, 1410s, 1327w, 1229s, 1192m, 1150s, 1121w, 1099m, 1042m, 1022m, 943m, 914m, 870s, 851m, 812m, 773s, 706s; ^1^H-NMR (DMSO-*d*_6_) *δ*_H_ 8.47–8.43 (2H, m, *H*-2 and 6), 7.65 (1H, ddd, *J* 8.3, 2.6, 1.5, *H*-4), 7.51 (1H, dd, *J* 8.1, 4.8, *H*-5); ^13^C-NMR (DMSO-*d*_6_) *δ*_C_ 161.9 (s), 147.3 (s), 146.8 (d), 146.6 (s), 141.5 (d), 126.3 (d), 124.6 (d); MS *m/z* (EI) 231 (M^+^+2, 17%), 229 (M^+^, 42), 168 (23), 130 (6), 125 (8), 104 (9), 102 (5), 93 (6), 78 (38), 70 (11), 64 (S_2_, 100), 51 (47).

*(Z)-3-(4-Chloro-5H-1,2,3-dithiazol-5-ylideneamino)pyridin-2-ol* (**1h**): Orange dust (284 mg, 53%), mp 151–152 °C (from pentane/DCM); Anal. found C, 34.3; H, 1.7; N, 17.0. C_7_H_4_ClN_3_OS_2_ requires C, 34.2; H, 1.6; N, 17.1%); UV *λ*_max_ (DCM)/nm 228 (log *ε* 2.75), 251 (2.67), 326 (2.64), 347 (2.64), 409 (2.77); IR *v*_max_/cm^−1^ 3125w (OH), 2922w, 2853w, 1638s, 1612m, 1572m, 1547m, 1524w, 1472m, 1433w, 1358w, 1346w, 1323w, 1310w, 1245m, 1236m, 1175w, 1144m, 1057w, 1020w, 959w, 943m, 910m, 893w, 858s, 826w, 797w, 779w, 770s; ^1^H-NMR (DMSO*-d*_6_) *δ*_H_ 12.07 (1H, br s, O*H*), 7.37–7.34 (2H, m, Py *H*), 6.33 (1H, dd, *J* 6.7, 6.7, Py *H*); ^13^C-NMR (DMSO*-d*_6_) *δ*_C_ 157.5 (s), 155.1 (s), 147.0 (s), 137.9 (s), 132.7 (d), 131.2 (d), 105.6 (d); MS *m/z* (EI) 247 (M^+^+2, 9%), 245 (M^+^, 21), 210 (32), 146 (100), 135 (9), 120 (13), 117 (10), 102 (12), 92 (40), 76 (11), 70 (33), 64 (77), 52 (19).

*(Z)-2-Chloro-N-(4-chloro-5H-1,2,3-dithiazol-5-ylidene)pyridin-3-amine* (**1i**): Yellow-orange needles (489.5 mg, 85%), mp 133–134 °C (from cyclohexane); Anal. found: C, 31.9; H, 1.0; N, 15.9. C_7_H_3_Cl_2_N_3_S_2_ requires C, 31.8; H, 1.1; N, 15.9%); UV *λ*_max_ (DCM) 232 (log *ε* 2.87), 281 (2.46), 363 (2.63); IR *v*_max_/cm^−1^ 3051w, 1722w, 1703w, 1657w, 1584s, 1566w, 1557w, 1506m, 1443w, 1400s, 1267w, 1250w, 1240w, 1207m, 1157m, 1082s, 1067w, 972w, 926w, 903w, 870s, 797m, 779s, 743s, 710s; ^1^H-NMR (CDCl_3_) *δ*_H_ 8.26 (1H, d, *J* 3.6, *H*-6), 7.45 (1H, dd, *J* 7.7, 1.3, *H-*4), 7.33 (1H, dd, *J* 7.8, 4.7, *H*-5); ^13^C-NMR (CDCl_3_) *δ*_C_ 162.8 (s), 147.3 (s), 146.4 (d), 145.0 (s), 142.5 (s), 127.3 (d), 123.4 (d); MS *m/z* (EI); 267 (M^+^+4, 4%), 265 (M^+^+2, 21), 263 (M^+^, 30), 204 (14), 202 (34), 164 (9), 135 (6), 125 (7), 112 (13), 103 (14), 93 (7), 76 (30), 70 (10), 64 (100), 50 (14).

*(Z)-4-Chloro-N-(4-chloro-5H-1,2,3-dithiazol-5-ylidene)pyridin-3-amine* (**1j**): Yellow prisms (437.6 mg, 76%), mp 160–161 °C (from cyclohexane/EtOH); Anal. found: C, 31.9; H, 1.1; N, 15.8. C_7_H_3_Cl_2_N_3_S_2_ requires C, 31.8; H, 1.1; N, 15.9%); UV *λ*_max_ (DCM) 231 (log *ε* 2.80), 279 (2.32), 364 (2.59); IR *v*_max_/cm^−1^ 3080w, 3057w, 1591s, 1557m, 1547w, 1506m, 1485w, 1466m, 1402m, 1283m, 1242m, 1225w, 1215w, 1146m, 1092s, 1053w, 966w, 912m, 862s, 831s, 783s, 739w, 708s; ^1^H-NMR (DMSO-*d*_6_) *δ*_H_ 8.48 (1H, s, *H*-2), 8.40 (1H, d, *J* 5.4, *H*-5 or 6), 7.70 (1H, d, *J* 5.4, *H*-5 or 6); ^13^C-NMR (DMSO-*d*_6_) *δ*_C_ 164.5 (s), 147.4 (d), 145.7 (s), 145.6 (s), 140.6 (d), 133.4 (s), 125.1 (d); MS *m/z* (EI) 267 (M^+^+4, 3%), 265 (M^+^+2, 20), 263 (M^+^, 26), 204 (9), 202 (21), 170 (3), 164 (3), 138 (3), 125 (7), 112 (12), 103 (9), 93 (5), 85 (12), 76 (18), 70 (7), 64 (100), 50 (15).

*(Z)-*N*-(4-Chloro-5*H*-1,2,3-dithiazol-5-ylidene)pyridin-4-amine* (**1k**): Yellow prisms (120 mg, 24%), mp 166–167 °C (from cyclohexane/EtOH); Anal. found: C, 36.5; H, 1.8; N, 18.1. C_7_H_4_ClN_3_S_2_ requires C, 36.6; H, 1.8; N, 18.3%); UV *λ*_max_ (DCM) 230 (log *ε* 2.82), 374 (2.63); IR *v*_max_/cm^−1^ 1593m, 1568s, 1549m, 1501m, 1485w, 1418m, 1246w, 1207m, 1152m, 1090w, 1053w, 999m, 872s, 858s, 827m, 779m, 735w; ^1^H-NMR (DMSO-*d*_6_) *δ*_H_ 8.63 (2H, d, *J* 5.1, *H*-2 and 6), 7.15 (2H, dd, *J* 4.7, 1.4, *H-*3 and 5); ^13^C-NMR (DMSO-*d*_6_) *δ*_C_ 162.5 (s), 158.1 (s), 151.6 (d), 146.4 (s), 113.9 (d); MS *m/z* (EI) 231 (M^+^+2, 34%), 229 (M^+^, 87), 194 (25), 168 (57), 162 (24), 130 (14), 127 (13), 125 (31), 104 (14), 93 (11), 78 (55), 64 (100), 51 (70).

*(Z)-N-(4-Chloro-5H-1,2,3-dithiazol-5-ylidene)-2,6-dimethylpyridin-4-amine* (**1l**): Yellow prisms (124 mg, 22%), mp 127–128 °C (from cyclohexane/EtOH); Anal. found: C, 42.0; H, 3.1; N, 16.3. C_9_H_8_ClN_3_S_2_ requires C, 41.9; H, 3.1; N, 16.3%); UV *λ*_max_ (DCM) 331 (log *ε* 3.01), 248 inf (2.72), 317 inf (2.28), 370 (2.75); IR *v*_max_/cm^−1^ 2920w, 1645w, 1584s, 1557m, 1508m, 1454w, 1410w, 1373w, 1319m, 1292w, 1269w, 1238w. 1207w, 1171s, 1123w, 1024w, 995w, 939m, 887s, 864w, 839m, 779s, 756w, 733m; ^1^H-NMR (CDCl_3_) *δ*_H_ 6.65 (2H, s, *H*-2 and 6), 2.50 (6H, s, 2 × C*H*_3_); ^13^C-NMR (CDCl_3_) *δ*_C_ 160.8 (s), 160.0 (s), 159.0 (s), 147.4 (s), 109.8 (d), 24.5 (*C*H_3_); MS *m/z* (EI) 259 (M^+^+2, 36%), 257 (M^+^, 84), 244 (5), 242 (20), 224 (12), 222 (38), 196 (14), 190 (31), 132 (100), 125 (13), 106 (25), 91 (6), 77 (7), 64 (51), 51 (6).

*(Z)-4-[(4-Chloro-5H-1,2,3-dithiazol-5-ylidene)amino]pyrimidine-5-carbonitrile* (**1m**): Orange prisms (340 mg, 61%), mp 205–206 °C (from EtOH); Anal. found: C, 32.9; H, 0.8; N, 27.3. C_7_H_2_ClN_5_S_2_ requires C, 32.9; H, 0.8; N, 27.4%); UV *λ*_max_ (DCM) 230 (log *ε* 2.70), 268 (2.57), 318 inf (2.06), 376 inf (2.50), 395 (2.88), 414 (3.08), 435 (2.99); IR *v*_max_/cm^−1^ 2239w and 2230w (C≡N), 1565w, 1560w, 1537m, 1507m, 1459s, 1424s, 1412s, 1391s, 1282w, 1192m, 1178w, 1162w, 1106w, 950w, 923s, 876s, 823w, 816m, 787m, 774m; ^1^H-NMR (acetone*-d*_6_) *δ*_H_ 9.45 (1H, s, *H*-2 or 6), 9.28 (1H, s, *H*-2 or 6); ^13^C-NMR (acetone*-d*_6_) *δ*_C_ 166.4 (s), 163.1 (d), 162.3 (s), 159.2 (d), 150.9 (s), 114.6 (*C*≡N), 105.6 (*C*C≡N); MS *m/z* (EI) 257 (M^+^+2, 12%), 255 (M^+^, 27), 220 (87), 194 (9), 125 (10), 104 (11), 102 (8), 93 (8), 77 (55), 70 (12), 64 (100), 51 (17).

## 4. Conclusions

The reaction conditions for the synthesis of a series of [(4-chloro-5*H*-1,2,3-dithiazolyl-idene)amino]azines were optimized with respect to base, temperature and reaction time. The optimum conditions involved mixing the aminoazine with Appel salt **4** in DCM at room temperature for 1 h, followed by the addition of amine base (2 equiv.) and then an additional 2 h of stirring at room temperature. Thirteen *N*-heteroazinyl dithiazolimines were successfully synthesized. With 2-pyridylamines, the choice of base was less important than with the 3- and 4-pyridylamines. In these cases the use of trialkylamines such as Et_3_N and Hünig’s base often gave superior product yields.
